# Building Segmentation in Urban and Rural Areas with MFA-Net: A Multidimensional Feature Adjustment Approach

**DOI:** 10.3390/s25082589

**Published:** 2025-04-19

**Authors:** Zijie Han, Xue Li, Xianteng Wang, Zihao Wu, Jian Liu

**Affiliations:** Key Laboratory of Earthquake Geodesy, Institute of Seismology, China Earthquake Administration, Wuhan 430071, China; 15337136270@163.com (Z.H.); leexue1211@126.com (X.L.); wangxt0724@126.com (X.W.); wuzihcn@163.com (Z.W.)

**Keywords:** building extraction, U^2^-net, multiscale feature fusion, remote sensing imagery, semantic segmentation

## Abstract

Deep-learning-based methods are crucial for building extraction from high-resolution remote sensing images, playing a key role in applications like natural disaster response, land resource management, and smart city development. However, extracting precise building from complex urban and rural environments remains challenging due to spectral variability and intricate background interference, particularly in densely packed and small buildings. To address these issues, we propose an enhanced U^2^-Net architecture, MFA-Net, which incorporates two key innovations: a Multidimensional Feature Adjustment (MFA) module that refines feature representations through Cascaded Channel, Spatial, and Multiscale Weighting Mechanisms and a Dynamic Fusion Loss function that enhances edge geometric fidelity. Evaluation on three datasets (Urban, Rural, and WHU) reveals that MFA-Net outperforms existing methods, with average improvements of 6% in F1-score and 7.3% in IoU and an average increase of 9.9% in training time. These advancements significantly improve edge delineation and the segmentation of dense building clusters, making MFA-Net especially beneficial for urban planning and land resource management.

## 1. Introduction

Extracting buildings from high-resolution remote sensing images is vital for many applications, including emergency response and management of natural disasters [1], utilizing and analyzing land resources, and planning and developing smart cities [2]. With ongoing advancements in Earth observation technologies, the automated identification of buildings in high-resolution remote sensing imagery has emerged as a key research focus [3].

Traditional methods for building extraction from high-resolution remote sensing images can be generally divided into two main categories based on the classification scale: per-pixel classification schemes [4] and object-oriented analysis [5]. The former focus on individual pixels or their immediate neighbors, identifying building features through spectral similarity. Common techniques in this category include maximum likelihood classification, decision trees, random forests, and support vector machines [6,7,8]. However, these methods often generate significant noise due to both homogeneity and heterogeneity in remote sensing images [9].

Object-oriented analysis methods utilize homogeneous pixel blocks derived from image segmentation as fundamental units, classifying them based on a combination of spectral, shading, geometric, and other features [10]. This approach leverages the spatial information inherent in buildings, effectively mitigating the pretzel noise issue. Nevertheless, object-based methods are generally limited to extracting buildings that are small in area and simple in structure [11]. Furthermore, these methods are highly susceptible to human factors, making it challenging to extract buildings that are extensive in range and complex in form [12]. Consequently, traditional methods often fail to meet the demands of high-precision, high-performance, and fully automated building extraction.

The swift development of artificial intelligence technologies, like deep learning (DL) [13], has resulted in substantial progress in feature extraction using convolutional neural networks (CNNs) [14]. CNNs can automatically learn relevant features from input remote sensing images, thereby minimizing the influence of human factors inherent in traditional methods [15]. As a result, CNNs have become a preferred approach for tasks like feasibility prediction, classification extraction, and automatic feature identification, which include applications such as road extraction [16] and landslide susceptibility mapping [17]. These networks excel at capturing hierarchical features through interconnected layers like convolutional, pooling, and activation layers, facilitating the extraction of fine details from remote sensing data [18,19]. Despite the advancements, training deep CNN models on large-scale, high-resolution remote sensing datasets presents significant challenges regarding computational resources and extended training time [20]. The efficiency of CNNs, especially in building extraction, remains a critical issue, particularly when aiming to achieve high accuracy in complex urban landscapes [21]. The availability of large volumes of high-resolution remote sensing images has provided ample training data, enabling CNN-based methods to excel in data-driven approaches and enhancing the generalization ability of building extraction [22]. However, these methods often suffer from problems such as overfitting, particularly in highly variable urban environments, and fail to capture intricate details, such as small or partially occluded structures [23,24,25,26]. In this context, improving the accuracy and training efficiency of CNN models for building extraction is of utmost importance.

Despite the abundant spectral information available in high-resolution remote sensing images, automatic building extraction continues to face significant challenges due to spectral variability among buildings and complex background noise [27]. Consequently, there is an urgent need for precise and high-performance automated building extraction methods. U^2^-Net [28] has garnered considerable attention for its intricate “U-squared” architecture, which offers an effective solution for enhanced feature extraction through its nested U-shaped design, enabling the capture of multiscale information [29]. This architecture enhances the model’s ability to distinguish between foreground and background features and achieves faster training times than other deep learning models of similar complexity, making it especially advantageous for large-scale urban mapping tasks [30]. However, its full potential has not been fully realized when applied to urban landscapes in high-resolution remote sensing imagery, often being limited by edge blurring [31] and misclassification in complex urban terrains.

To address this issue, this study presents comprehensive enhancements to the U^2^-Net model, aiming to improve its specificity and reliability for building detection. We propose a Multidimensional Feature Adjustment (MFA) module that combines local detail enhancement with broader contextual understanding by incorporating Channel Weighting Mechanisms (CWM) [32], Spatial Weighting Mechanisms (SWM) [33], and a Multiscale Information Fusion (MSIF) step, which emphasizes critical features across multiple scales for effective detection in both congested urban and sparse rural areas. Additionally, a Dice-inspired loss function [34] penalizes false positives and improves edge accuracy [35], thereby optimizing the sensitivity-specificity trade-off for more precise building detection. We evaluated our method using self-built Urban and Rural datasets, as well as the publicly available WHU building dataset, and compared it with eight widely used benchmark models.

## 2. Materials and Methods

### 2.1. Study Area and Data

To assess the effectiveness of our proposed method, we utilized three building datasets: Urban dataset, Rural dataset, and the WHU building dataset [36]. The use of these diverse datasets enables a thorough evaluation of the model’s performance across a range of building types, densities, and environmental conditions, ensuring that the model is robust and adaptable to various real-world scenarios.

#### 2.1.1. Urban Dataset

The Urban dataset was selected to test the model’s performance in high-density urban environments. It covers selected areas of Wuhan, China, including both residential and commercial zones that feature densely packed, multistory buildings. These urban areas are characterized by a high concentration of buildings with complex, irregular shapes, often featuring intricate details such as balconies, courtyards, and connecting structures. The closely arranged buildings and the frequent presence of overlapping shadows and reflective surfaces create challenges for accurate edge detection and segmentation. The complexity of building layouts in these environments makes it essential for the model to be capable of distinguishing fine boundaries between adjacent structures, even when the edges are not well defined.

**Figure 1:**
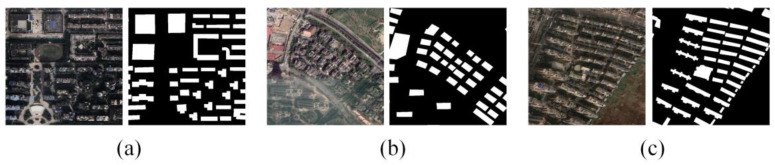
Sample images from the Urban dataset: (**a**–**c**) Original images (**left**) and corresponding labels (**right**).

The Urban dataset consists of 5536 images for training, 1542 for validation, and 184 for testing. All images have a spatial resolution of 0.5 m and were manually annotated to ensure accuracy. The dataset was divided into 7958 non-overlapping tiles, each measuring 640 × 640 pixels. Sample images from the Urban dataset are shown in Figure 1.

#### 2.1.2. Rural Dataset

The Rural dataset was chosen to assess the model’s ability to handle low-density, scattered building structures in rural environments. This dataset focuses on peri-urban and rural villages surrounding Wuhan, characterized by low-rise buildings that are dispersed over a wide area. The buildings in this dataset are generally less uniform in shape compared to their urban counterparts, often irregular in structure and interspersed with natural elements like vegetation, fields, and agricultural landscapes. The scattering of buildings, along with occlusions caused by trees and other obstacles, presents significant challenges for the model, particularly when it comes to capturing the fine edges of structures and dealing with background clutter.

**Figure 2:**
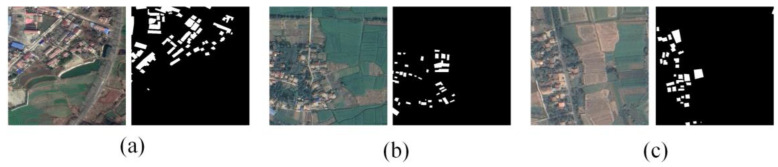
Sample images from the Rural dataset: (**a**–**c**) Original images (**left**) and corresponding labels (**right**).

The Rural dataset includes 530 images for training, 134 for validation, and 32 for testing, all with the same 0.5 m spatial resolution. Like the Urban dataset, the images were manually annotated, and the imagery was segmented into 640 × 640-pixel tiles. Sample images from the Rural dataset are shown in Figure 2.

#### 2.1.3. WHU Building Dataset

The WHU building dataset, an internationally recognized open-source dataset, was included to assess the model’s ability to generalize to different geographical regions and building types. This dataset contains aerial imagery of Christchurch, New Zealand, providing a diverse set of building structures in urban and suburban environments that differ significantly from the self-collected Urban and Rural datasets. The WHU dataset allows for comparison of model performance across different geographical areas, offering valuable insights into how well the model can adapt to architectural styles and building distributions in regions outside of China.

**Figure 3:**
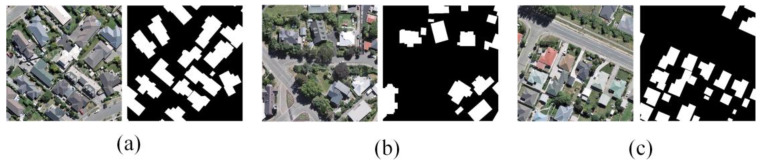
Sample images from the WHU building dataset: (**a**–**c**) Original images (**left**) and corresponding labels (**right**).

The WHU dataset consists of 8188 images, each sized 512 × 512 pixels, and covers an area of 450 km in Christchurch. The images have been downsampled to resolutions ranging from 0.075 to 0.300 m. The dataset is divided into 9420 images for training, 1537 for validation, and 3848 for testing. By using this dataset, we can evaluate the model’s performance in a setting with a different architectural style, as well as test its robustness in regions with distinct environmental factors, such as different levels of urbanization and climate conditions. This international dataset complements the self-collected Urban and Rural datasets and enables a broader evaluation of the model’s generalization capability. Sample images from the WHU building dataset are shown in Figure 3.

### 2.2. Method

#### 2.2.1. Overview of the Model

**Figure 4:**
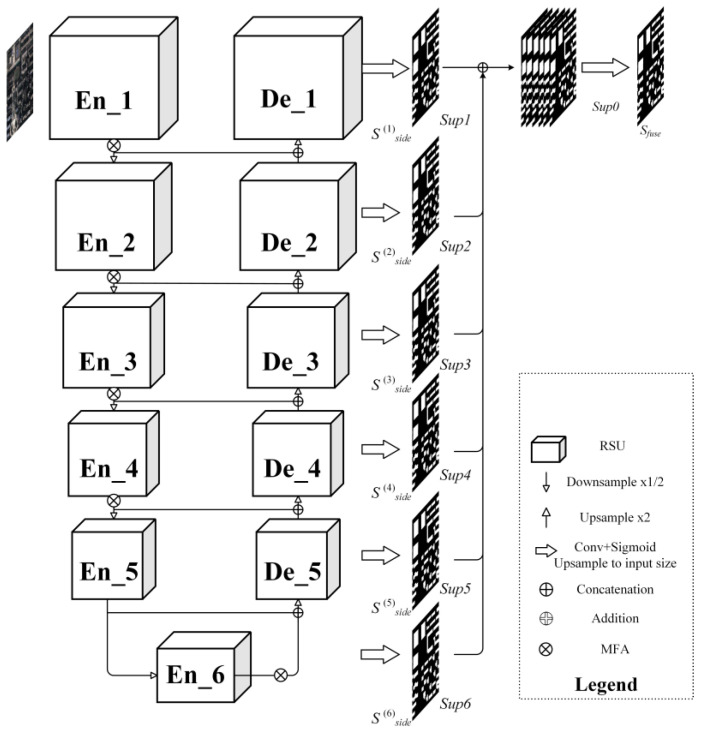
Illustration of the overall framework of the proposed model.

U^2^-Net utilizes a two-tiered nested U-shaped architecture. The primary layer features an extensive U-shaped framework comprising 11 stages, each incorporating a residual U-shaped block (RSU) as its secondary component. This layered U-shaped configuration is designed to enhance the efficient extraction of multiscale and hierarchical features. The overall, illustrated in Figure 4, is organized into three main sections: (1) the encoder, (2) the decoder, and (3) the map fusion block, which are further detailed below.

**Figure 5:**
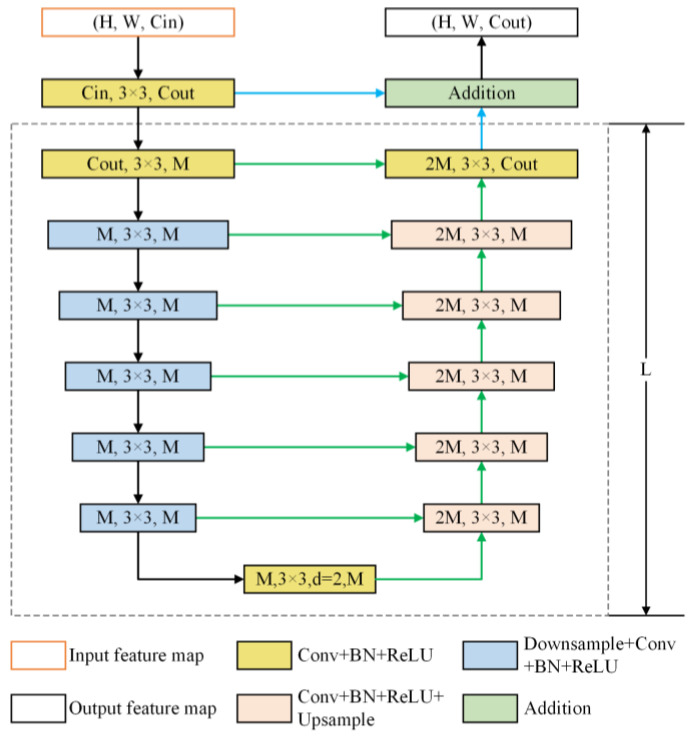
Residual U-Block.

Encoder stage. The encoder comprises six stages, integrating a Residual U-shaped (RSU) block. In the first four RSU stages, feature maps are progressively downsampled to enlarge the receptive field and capture information at broader scales. For the last two stages, dilated convolutions replace pooling operations, allowing the preservation of contextual information while maintaining the original feature map size despite the increased receptive field. The structure of the RSU block is shown in Figure 5.

Decoder stage. Mirroring the encoder stage, the decoder also consists of six stages. Upsampled feature maps from the previous decoder layer are merged in each decoder stage with the corresponding feature maps from the symmetric encoder stage. This integration facilitates the combination of multiscale information, ensuring accurate reconstruction of spatial details and enhancing the overall feature representation.

Map fusion block. The final component integrates feature maps through a deeply supervised approach to produce a probabilistic output. The model generates six side outputs, which are subsequently upsampled to match the dimensions of the input image and combined sequentially.

The U^2^-Net model integrates a deep architecture with strong multiscale capabilities while keeping computational and memory requirements low. Additionally, because U^2^-Net is made up exclusively of RSU blocks and does not depend on any pre-trained backbone, it provides significant flexibility. This allows it to be easily adjusted to various tasks with minimal loss in performance. Initially, the convolutional layer processes the input feature map $\left(H,W,\;and\;C\right)$, converting it into an intermediate map, $F\left(x\right)$, with channel X. This layer is designed to capture local features. Then, a symmetric encoder–decoder with depth L processes the intermediate feature map, $F\left(x\right)$, enabling the model to capture multiscale information and minimize the loss of contextual details during upsampling. Lastly, local and multiscale features are integrated through residual concatenation.

#### 2.2.2. MFA Module

In this study, we propose an integrated Multidimensional Feature Adjustment (MFA) module that combines the Weighting Mechanism and the Multiscale Information Fusion module to enhance the model’s ability to perceive multiscale features and global contextual information. The MFA module adopts an encoder–decoder architecture to improve feature extraction accuracy and overall model performance. The MFA module is inserted after six encoding stage in the U^2^-Net architecture (Figure 4).

The overall process of the MFA module is as follows:

1. The input image first enters the network and undergoes a series of encoding layers to produce a feature map. This feature map then passes through the MFA module. Within the MFA module, the feature map is first processed by the “Channel Weighting Mechanism”. The Channel Weighting Mechanism (CWM) adaptively adjusts channel-wise importance within the feature map. To do this, max pooling and average pooling operations are applied to the input feature map. The results of these pooling operations are then passed through a shared “Feature Reconstruction Layer” *MLP*. The *MLP* performs compression and reconstruction of the pooled features, producing a set of weights that represent the importance of each channel. These weight coefficients, generated using the Sigmoid activation function, are applied to each channel of the input feature map, either enhancing or suppressing the channel’s contribution based on its importance. The final output of the Channel Weighting Mechanism is a feature map where each channel has been weighted according to its significance.

The process can be described by the following equation:

(1) $$x_{CWM}=x\cdot \sigma \left(\sum_{i=1}^{N}MLP\left({pool}_{i}\left(x_{in}\right)\right)\right)$$

where $x_{in}$ is the input feature map, ${pool}_{i}\left(x_{in}\right)$ represents different pooling operations (e.g., average pooling and max pooling), MLP denotes a multilayer perceptron, and σ is the Sigmoid activation function.

**Figure 6:**
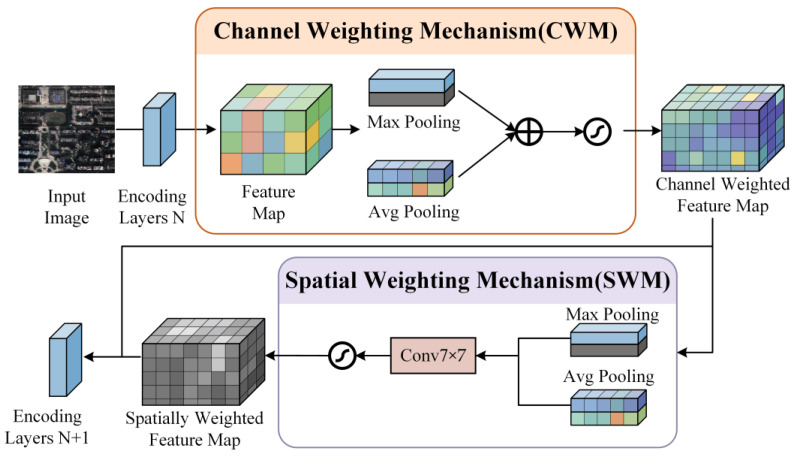
Structure of the CWM module and SWM module.

2. Spatial Weighting Mechanism: Next, the feature map enters the “Spatial Weighting Mechanism”, which aims to enhance the focus on more important spatial locations within the image. The Spatial Weighting Mechanism begins by applying pooling operations along the channel dimension, resulting in two separate pooled feature maps: one obtained through max pooling and the other through average pooling. These two pooled feature maps are concatenated along the channel dimension, then passed through a convolutional layer to generate a spatial attention map. This map indicates the significance of each spatial location within the feature map. The Sigmoid activation function is applied to this map, generating a weight coefficient. This coefficient is then element-wise multiplied with the channel-refined feature map, enabling the model to emphasize important spatial regions. The final output is a spatially weighted feature map that accentuates the most relevant areas for the task. The overall structure of the Channel Weighting Module (CWM) and the Spatial Weighting Module (SWM) is shown in Figure 6.

The Spatial Weighting Mechanism can be expressed as follows:

(2) $$x_{SWM}=x_{CWM}\cdot \sigma ({Conv}_{7\times 7}\;(ChannelPool(x_{CWM})))$$

where $ChannelPool(x_{CWM})$ denotes the pooling operations along the channel dimension, and Conv represents the convolution operation.

**Figure 7:**
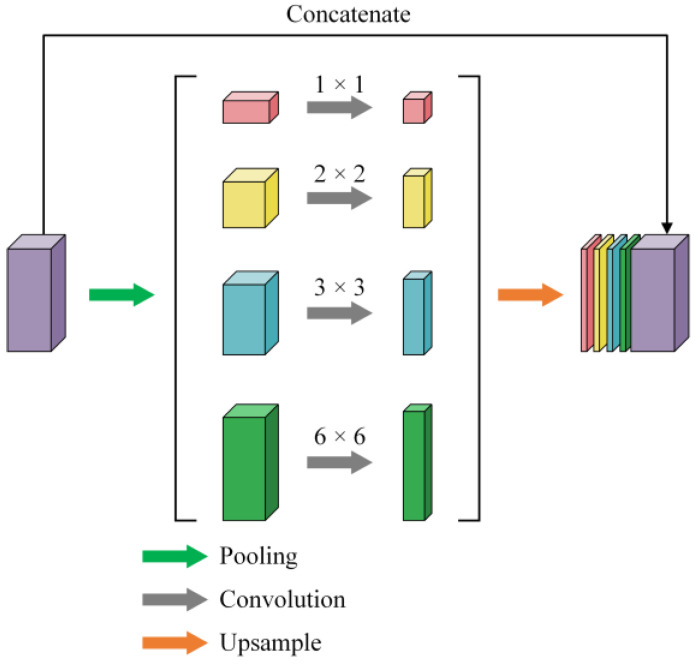
Structure of the MSIF module.

3. Multiscale Information Fusion Module: Finally, the feature map enters the “Multiscale Information Fusion Module” (MSIF), which enhances the model’s ability to capture global contextual information at multiple scales. The MSIF module employs adaptive average pooling at four scales (1 × 1, 2 × 2, 3 × 3, and 6 × 6) to capture hierarchical contextual patterns. Each pooled feature map is then processed by a 1 × 1 convolutional layer for channel reduction before upsampling. These pooled feature maps are passed through convolutional layers to adjust the number of channels, ensuring that each feature map, regardless of its scale, has a consistent channel count. Afterward, the pooled feature maps are upsampled using bilinear interpolation to match the spatial dimensions of the original input feature map. The upsampled feature maps are then concatenated along the channel dimension, producing a multiscale feature map that integrates contextual information from all scales. This process enables the model to extract and fuse global contextual information, improving its understanding of the image at different levels of detail. The overall structure of the MSIF module is shown in Figure 7.

The Multiscale Information Fusion module can be described as follows:

(3) $$x_{\mathrm{MSIF}}=\mathrm{Concat}\left\{{\mathrm{Upsample}}_{k}\left({\mathrm{Conv}}_{k}\left({\mathrm{AdaptiveAvgPool2d}}_{k}(x)\right)\right)\right\},{}^{\circ}k\in \{1,2,3,6\}$$

where AdaptiveAvgPool2d(x) performs pooling at different scales, Conv reduces the number of channels, and Upsample restores the pooled feature maps to the original spatial dimensions.

**Figure 8:**
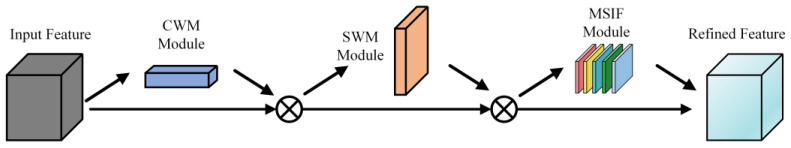
Structure of the MFA module.

MFA differs from CBAM in three main aspects while offering notable advantages through the synergy among its three components. First, whereas CBAM applies a sequential channel-and-spatial attention approach, MFA incorporates an additional multiscale stage that captures localized features and broader contextual cues across multiple resolutions. By processing features through three stages, MFA filters out less informative channels, highlights critical spatial regions, and gathers multiscale information to handle objects or regions of varying sizes. Second, MFA is specifically integrated into an encoder–decoder architecture (U^2^-Net) to leverage multiscale downsampling and upsampling operations. In contrast, CBAM is typically inserted into standard classification backbones without explicitly aggregating context from different scales. Third, MFA merges channel, spatial, and multiscale cues in one unified pipeline, creating richer, more balanced input data for subsequent layers. In contrast, CBAM processes channel and spatial attention in sequence and does not have a dedicated multiscale component, potentially limiting its capacity to address large-scale variations. This three-stage synergy improves accuracy in local detail and offers global contextual awareness, ultimately enhancing the network’s reliability in diverse situations. Specifically, after the CWM refines channel importance, the SWM pinpoints which spatial regions are most relevant, and then the MSIF aggregates contextual clues from multiple scales. The result is a set of enhanced feature maps that enable more consistent decision-making, offering fine-grained accuracy and broader contextual understanding essential for complex vision tasks. The overall structure of the MFA module is shown in Figure 8.

The specific formulation of the MFA module is as follows:

(4) $$x_{\mathrm{MFA}}=x_{\mathrm{CWM}}\cdot x_{\mathrm{SWM}}\cdot x_{\mathrm{MSIF}}$$

#### 2.2.3. Dynamic Fusion Loss

To address the dual challenges of pixel-wise classification accuracy and structural coherence in building extraction, we propose a Dynamic Fusion Loss ($L_{DF}$) that intelligently balances two complementary loss functions: binary cross-entropy (BCE) [37] and Dice loss functions [34]. Unlike conventional static loss combinations, our approach implements an epoch-adaptive weighting strategy that dynamically shifts focus between fine-grained pixel classification and holistic structural optimization during training. The composite loss is formulated as follows:

(5) $$L_{DF}=\alpha_{bce}\cdot L_{BCE}+\alpha_{dice}\cdot L_{Dice}$$

The weighting coefficients undergo linear progression across training epochs. Given the model output O and the ground truth labels T, as well as the current training epoch count $E_{current}$, the total number of epochs $E_{total}$, the initial Dice loss weight $\alpha_{init}$, the final Dice loss weight $\alpha_{final}$, the Dice loss weight $\alpha_{dice}$, and BCE loss weight $\alpha_{bce}$, at each training epoch, are determined as follows:

(6) $$\alpha_{dice}=\alpha_{init}+\left(\alpha_{final}-\alpha_{init}\right)\cdot \frac{E_{current}}{E_{total}}$$

(7) $$\alpha_{bce}=1-\alpha_{dice}$$

The BCE loss is defined as follows:

(8) $$L_{BCE}=-\frac{1}{HW}\sum_{i=1}^{H}\sum_{j=1}^{W}\left[T_{ij}log\left(\sigma \left(O_{ij}\right)\right)+\left(1-T_{ij}\right)log\left(1-\sigma \left(O_{ij}\right)\right)\right]$$

where H and W represent the height and width of the image, $T_{ij}$ is the true label at position $\left(i,j\right)$, and $O_{ij}$ is the model output at position $\left(i,j\right)$. $\sigma \left(.\right)$ is the sigmoid activation function, which converts the model output into probabilities. This term penalizes classification errors at individual pixels, crucial for mitigating false positives in complex scenes.

The Dice loss is defined as follows:

(9) $$L_{Dice}=1-\frac{2\sum_{i=1}^{H}\sum_{j=1}^{W}\left(\sigma \left(O_{ij}\right)\cdot T_{ij}\right)+\epsilon }{\sum_{i=1}^{H}\sum_{j=1}^{W}\sigma \left(O_{ij}\right)+\sum_{i=1}^{H}\sum_{j=1}^{W}T_{ij}+\epsilon }$$

The smoothing factor $\epsilon =1\times 10^{-6}$ prevents division instability while maintaining gradient validity for small objects. By optimizing the overlap between predicted and ground-truth regions, this term enhances connectivity for fragmented buildings and suppresses “salt-and-pepper” noise.

In our implementation, we set $\alpha_{final}=0.9$ based on empirical experiments across multiple validation datasets. During preliminary trials, we tested different initial and final weights (e.g., $\alpha_{init}$ in $\left[0.1,0.3\right]$ and $\alpha_{final}$ in $\left[0.7,1.0\right]$) and observed that starting with a low Dice weight ($\alpha_{init}=0.1$) facilitated more stable early training by relying on pixel-wise BCE guidance, while gradually shifting to a higher Dice weight improved structural coherence in later epochs. Although these specific values consistently yielded favorable results in terms of both segmentation accuracy and boundary smoothness, we do not claim they are universally optimal for all datasets or tasks.

The dynamic weighting strategy follows a linear adjustment mechanism, where the Dice loss weight A gradually increases from a small initial value (e.g., 0.1) to a larger final value (e.g., 0.9) over the course of training. This smooth transition enables the model to focus on pixel-level classification accuracy in the early epochs—primarily guided by the BCE loss—and progressively shifts toward region-level structural coherence, where the Dice loss becomes more dominant. By continuously balancing these two objectives throughout the training process, the strategy improves the model’s ability to capture fine-grained details while also preserving the overall shape and connectivity of building regions. This design also helps mitigate class imbalance, particularly in rural scenes where building distributions are sparse.

### 2.3. Experiments Setup

The model was developed and tested using 4 × NVIDIA Tesla V100 (32GB VRAM) with PyTorch 2.0.1. We employed the AdamW optimizer [38] with an initial learning rate of 0.001, β₁ set to 0.9, and ε set to 10^−8^. The training process was carried out for 550 epochs, using a batch size of 16. The images were first resized to a fixed base size, followed by random cropping to introduce variability in the region of interest. Next, the images were normalized using dataset-specific mean and standard deviation values to maintain consistent pixel value distribution throughout training. These preprocessing steps were followed by feeding the processed images into the model. After each upsampling stage, the feature map was resized to the original image dimensions using bilinear interpolation, resulting in six side outputs. These outputs were concatenated along the channel dimension to create a feature map matching the input image size, with six channels. The final fusion result was generated through a convolutional layer, and the seven outputs were used along with the ground truth labels to compute the loss, which was then used for backpropagation and parameter optimization.

In terms of experimental design, we evaluated the performance of eight different models across three building datasets: Urban, Rural, and WHU building datasets. The eight models included traditional architectures (LR-ASPP, FCN, PSPNet, DeepLabv3, DeepLabv3+, and UNet), the baseline model U^2^-Net proposed in this study, and the final improved model MFA-Net. Each model was tested under identical conditions to ensure fair comparison. To assess the contribution of various modules, we established multiple experimental configurations: the baseline model, U^2^-Net + M (MFA), U^2^-Net + D (Dynamic Fusion Loss), and U^2^-Net + M + D. These configurations were rigorously tested on all three datasets, allowing us to verify the effectiveness of the proposed improvements. This experimental setup facilitates a comparative analysis of different strategies for improving building extraction performance and provides solid experimental evidence for future research and practical applications.

### 2.4. Evaluation Metrics

The model produces a probability map with the same spatial resolution as the input image, assigning each pixel in the prediction map a value between 0 and 1. The ground truth is usually represented as a binary mask, where each pixel is labeled as either 0 or 1 (1 indicating building or foreground pixels and 0 indicating background). To evaluate the performance of our method, which can be viewed as a variation of the semantic segmentation task, we use four widely adopted metrics in traditional semantic segmentation: Intersection over Union (IoU), Recall, Precision, and F1. In addition to calculating the average values of these metrics using the validation set, we also compute the metrics for each individual image in the validation set. Furthermore, we calculate the standard deviation (std) for each of these four metrics across the entire validation set, providing an indication of the model’s consistency in performance.

IoU. This metric measures the ratio of the intersection to the union of the predicted and ground truth masks (Equation (8)).

Precision. Precision quantifies the proportion of correctly predicted “building” pixels relative to all pixels that the model predicted as “building” (Equation (9)).

Recall. Recall measures the proportion of “building” pixels in the ground truth that were correctly identified by the model (Equation (10)).

F1. The F1 is the harmonic mean of the Precision and Recall, providing a balanced measure of the model’s accuracy [39] (Equations (10)–(13)).

(10) $$IoU=\frac{TP}{TP+FP+FN}$$

(11) $$Precision=\frac{TP}{TP+FP}$$

(12) $$Recall=\frac{TP}{TP+FN}$$

(13) $$F1=\frac{2\times Precision\times Recall}{Precision+Recall}$$

where

True positive (*TP*): The number of pixels correctly identified as belonging to the “building” category.

False negative (*FN*): The number of “building” pixels in the ground truth that were not detected by the model.

False positive (*FP*): The number of “non-building” pixels incorrectly labeled as “building” by the model.

True negative (*TN*): The number of “non-building” pixels correctly identified as such by the model.

## 3. Experimental Results

### 3.1. Experiments on the Urban Building Dataset

**Figure 9:**
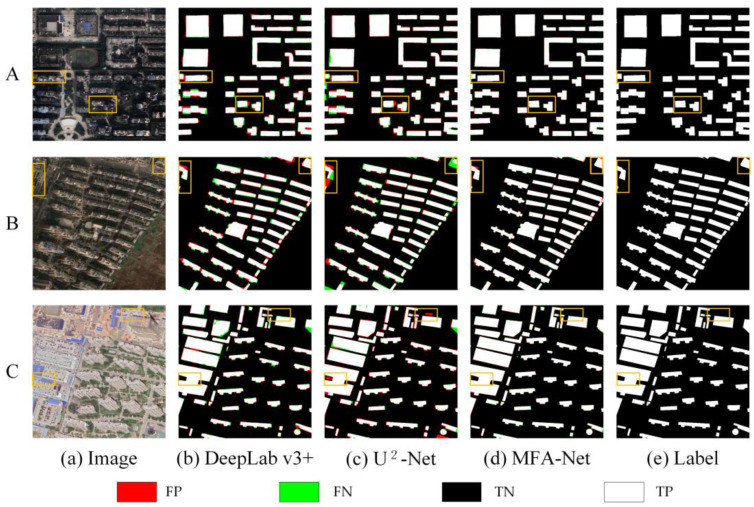
Comparison of prediction results of different models on the Urban dataset. A, B, and C represent different urban areas selected from the dataset.

**Figure A1:**
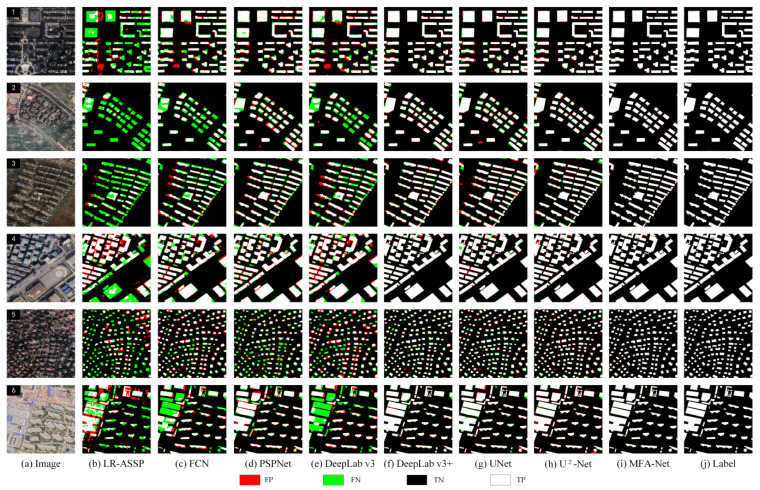
Comparison of prediction results of 8 models on the Urban dataset.

As shown in Figure 9, MFA-Net outperformed other models, including the baseline U^2^-Net, across multiple evaluation metrics. It achieved a higher F1 score than U^2^-Net, indicating its ability to balance Precision and Recall while preserving overall structural integrity in building extraction. It also demonstrated better results in Precision, Recall, and IoU, surpassing U^2^-Net by comparable margins. For a comprehensive comparison of all eight methods across the Urban dataset, including detailed regional performance analyses, see Figure A1 and Table A1 in Appendix A.

When examining performance stability, MFA-Net showed lower variability in its results than U^2^-Net, reflecting more consistent outcomes across different samples in the validation set.

MFA-Net required more training time compared to U^2^-Net. As shown in Table 1, although this increase may appear noteworthy, the additional computational cost could be justified in large-scale applications where higher accuracy is crucial. The improved segmentation quality suggests a reduced likelihood of false positives and negatives, which can be especially valuable in real-world scenarios, offering enhanced accuracy with relatively efficient training for urban building extraction tasks.

### 3.2. Experiments on the Rural Building Dataset

**Figure 10:**
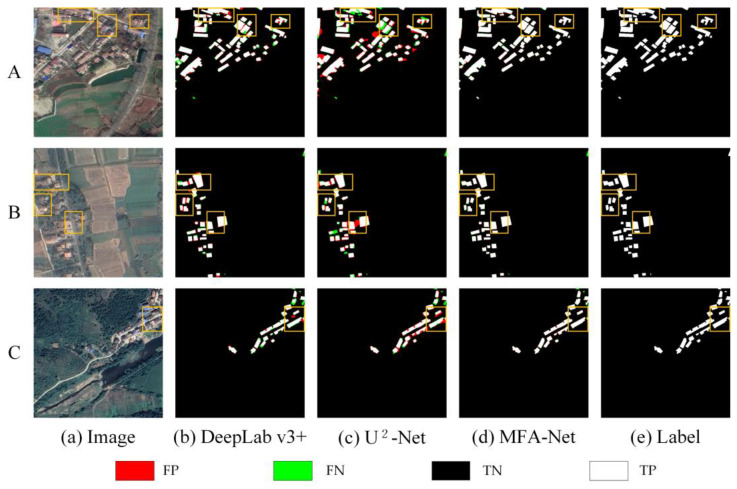
Comparison of prediction results of different models on the Rural dataset. A, B, and C represent different rural areas selected from the dataset.

Experimental results on the Rural Building dataset (Figure 10) indicate that MFA-Net achieves improved accuracy and robustness in complex rural environments. It effectively minimizes both false and missed detections, indicating its capability to handle irregularly shaped structures where previous models, including U^2^-Net, may face challenges.

**Figure A2:**
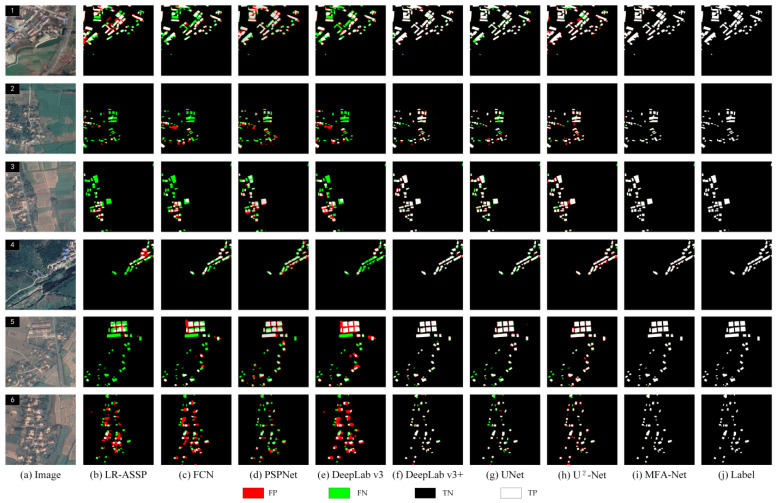
Comparison of prediction results of 8 models on the Rural dataset.

According to Table 2, MFA-Net surpasses U^2^-Net in F1, Precision, Recall, and IoU by considerable margins, with lower standard deviations suggesting more stable and consistent performance. For a comprehensive comparison of all eight methods across the Rural dataset, including detailed regional performance analyses, see Figure A2 and Table A2 in Appendix A.

Although MFA-Net’s training time is longer than U^2^-Net’s, the observed improvements in performance may justify the additional computational cost for applications requiring higher accuracy. The enhancements in Precision, Recall, and IoU suggest that MFA-Net is better suited for the complexities of rural building extraction.

### 3.3. Experiments on the WHU Building Dataset

**Figure A3:**
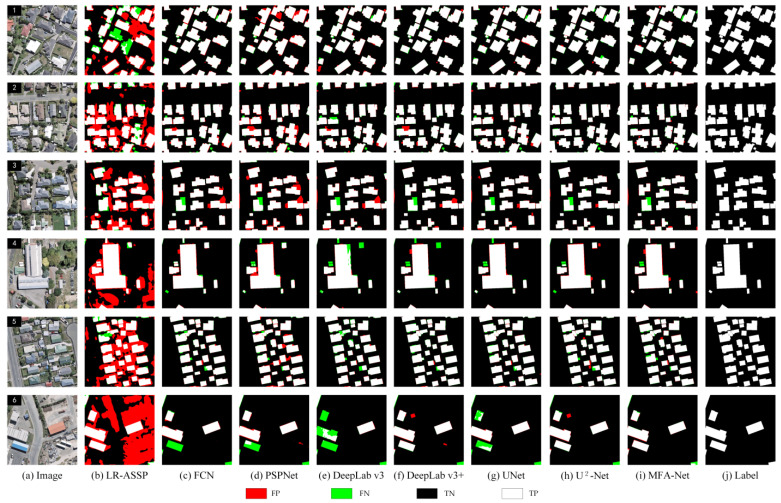
Comparison of prediction results of 8 models on the WHU Building dataset.

To further validate MFA-Net’s performance on diverse datasets, we tested it on the WHU Building dataset and compared the results with U^2^-Net. For a comprehensive comparison of all eight methods across the WHU Building dataset, including detailed regional performance analyses, see Figure A3 and Table A3 in Appendix A.

**Figure 11:**
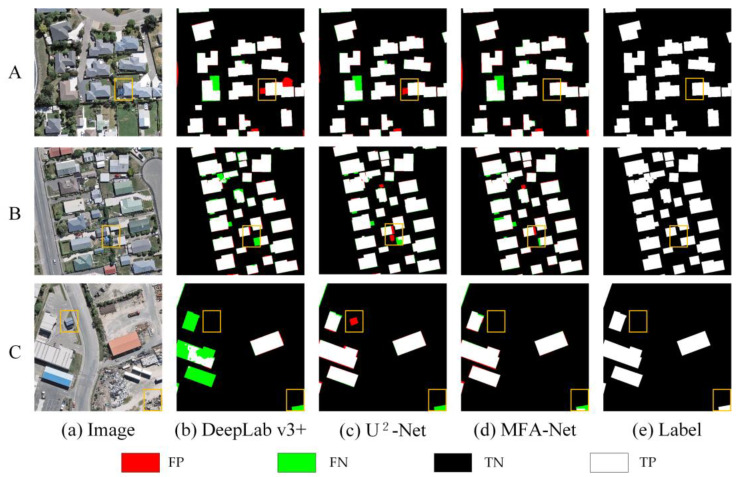
Comparison of prediction results of different models on the WHU Building dataset. A, B, and C represent different areas selected from the WHU dataset.

As shown in Figure 11, MFA-Net delineates building edges more accurately and maintains overall structural integrity. According to Table 3, MFA-Net surpasses U^2^-Net in F1, Precision, Recall, and IoU, with lower standard deviations in certain metrics, indicating more consistent performance. Although MFA-Net’s training time is slightly longer, the enhanced accuracy and robustness may justify the additional computational cost in scenarios requiring precise building extraction.

### 3.4. Ablation Experiments

To evaluate how the M (MFA) and D (Dynamic Fusion Loss) modules affect model performance, ablation experiments were conducted on the Urban, Rural, and WHU Building datasets. U^2^-Net served as the baseline, and the M and D modules were progressively incorporated to assess their respective contributions.

#### 3.4.1. Ablation Experiments on the Urban Dataset

**Figure 12:**
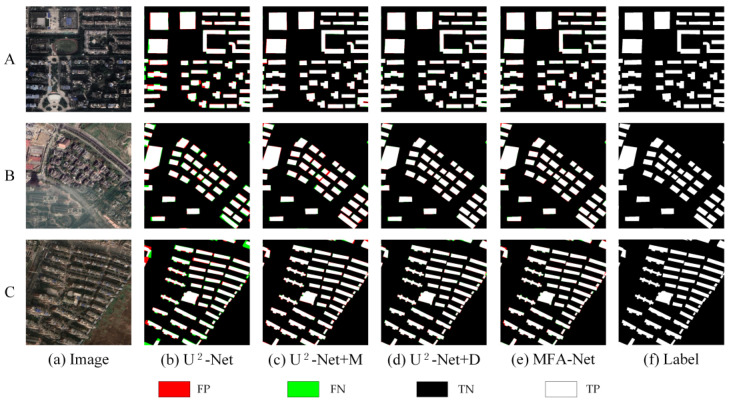
Comparison of ablation experiment results on the Urban dataset. A, B, and C represent different urban areas selected from the dataset.

On the Urban dataset, the baseline U^2^-Net provided an initial level of performance. Introducing the M module (U^2^-Net + M) improved F1, Precision, and Recall. Similarly, adding the D module (U^2^-Net + D) also enhanced these metrics, indicating that balancing positive and negative samples benefits overall accuracy. As shown in Figure 12 and Table 4, combining both modules (U^2^-Net + M + D, i.e., MFA-Net) yielded the highest performance.

#### 3.4.2. Ablation Experiments on the Rural Dataset

**Figure 13:**
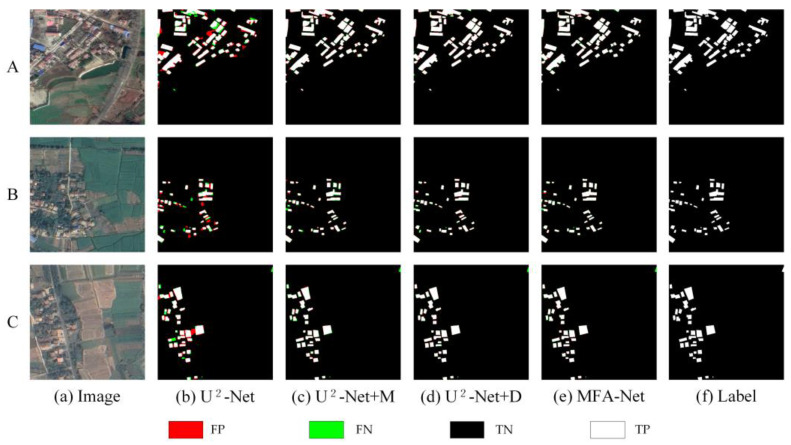
Comparison of ablation experiment results on the Rural dataset. A, B, and C represent different rural areas selected from the dataset.

On the Rural dataset, a similar trend emerged. Starting with U^2^-Net, adding the M module noticeably improved the model’s ability to detect buildings with varied textures. Including the D module further enhanced F1, Precision, and Recall, and the combined approach (U^2^-Net + M + D) achieved the best results in rural building extraction, as illustrated in Figure 13 and Table 5.

#### 3.4.3. Ablation Experiments on the WHU Building Dataset

**Figure 14:**
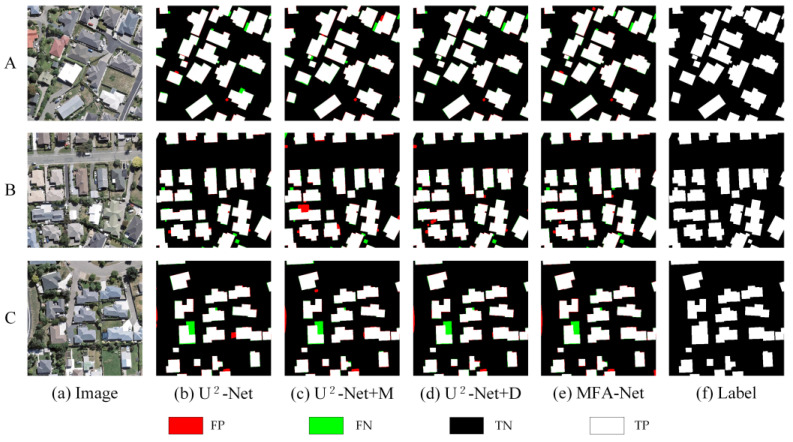
Comparison of ablation experiment results on the WHU Building dataset. A, B, and C represent different areas selected from the WHU dataset.

On the WHU Building dataset, U^2^-Net again served as the baseline. Adding the M module improved edge recognition and detail capture. Further incorporating the D module strengthened performance, and merging both modules (MFA-Net) provided the highest F1, Precision, and Recall among all tested configurations, as illustrated in Figure 14 and Table 6.

## 4. Discussion

This study thoroughly evaluated the performance of various semantic segmentation models across three diverse remote sensing image datasets: Urban, Rural, and WHU building dataset. The results clearly demonstrate that MFA-Net excels in all these environments, outperforming other models, including the baseline U^2^-Net, in terms of both precision and robustness. The following provide a detailed analysis of MFA-Net’s performance on each dataset, supported by the findings from the ablation experiments, which highlight the contributions of specific modules to the model’s success.

### 4.1. Algorithm Performance and Improvements

On the Urban dataset: MFA-Net significantly improved the accuracy and reliability of building extraction. Notably, the model enhanced the clarity of building edge information, reducing both false positives and false negatives along building edges (Figure 9, images A). This is particularly evident in densely packed areas, where MFA-Net effectively distinguished individual buildings, even in regions with complex structures or small gaps between buildings (Figure 9, images C). Additionally, the model was highly effective in identifying incomplete building structures. MFA-Net successfully captured the edge details of partially occluded buildings, reducing errors and improving detection even in areas where building boundaries are not fully visible (Figure 9, images B). The model’s ability to capture fine building contours was significantly enhanced, ensuring that edge details were more precise compared to other models. The integration of the enhanced focus mechanism through the MFA module, coupled with multilevel context aggregation, was crucial in improving the model’s ability to distinguish fine building details and accurately detect edges, especially in complex and densely packed urban environments. MFA-Net’s enhanced focus on critical regions, particularly the boundaries of tightly packed buildings, helped to overcome the challenges posed by complex backgrounds and spectral variability. Moreover, the dynamically weighted loss function further optimized the model’s ability to handle imbalanced positive and negative samples, contributing to superior edge detection precision.

On the Rural dataset: MFA-Net demonstrated its outstanding ability to address the challenges posed by the complex and varied rural environments, which often involve irregularly shaped buildings and a significant imbalance between foreground and background, where the background dominates the dataset. Despite these difficulties, MFA-Net maintained a high level of extraction accuracy, minimizing false positives and false negatives, even in scenarios where the foreground–background ratio was highly imbalanced. The model also improved the clarity of building edge information, reducing detection errors along the boundaries of buildings. Notably, MFA-Net excelled in extracting small buildings, which is often a challenging task in rural settings where buildings tend to be smaller and more dispersed (Figure 10). In crowded areas with densely packed small buildings, MFA-Net was able to clearly distinguish between individual structures, even in regions with complex structures or small gaps between buildings (Figure 10, images A and C). Additionally, MFA-Net proved to be highly effective in extracting information from irregularly shaped buildings, offering a more complete and accurate extraction than previous models (Figure 10, image A and B). These advancements can be attributed to the enhanced focus mechanism and multilevel context aggregation in MFA-Net, which allowed the model to adapt to the specific challenges of rural building extraction. Quantitative analysis further supported these findings, with MFA-Net showing significant improvements over U^2^-Net in all key metrics, including F1, Precision, Recall, and IoU. This confirms MFA-Net’s robustness and its suitability for rural building extraction tasks, where high accuracy is essential for practical applications.

On the WHU Building Dataset: MFA-Net demonstrated improvements over other models, particularly in capturing details in both regular and large building regions. The model showed advancements in reducing missed and false detections, especially compared to the baseline model U^2^-Net. MFA-Net effectively minimized false positives and missed detections common in other models, but MFA-Net was still able to minimize such errors effectively, particularly in complex building structures (Figure 11, images A, B, and C). Additionally, MFA-Net excelled in capturing incomplete building information at the edges of images—an area where other models often failed to detect (Figure 11, image C).

Although MFA-Net achieved notable performance gains on multiple datasets, its improvement on the WHU Building dataset was relatively smaller (e.g., a 1.3% increase in F1 score) compared to the 13.6% gain observed on our self-built Rural dataset. We believe there are several reasons for this pronounced difference:

Differences in Study Areas. The Rural dataset primarily features buildings with simpler outlines and more homogeneous surroundings, which may align better with MFA-Net’s architecture and training. In contrast, the WHU dataset encompasses a broader range of building shapes, rooftop materials, and contextual environments, making it more challenging for the model to generalize effectively.

Broader Data Distribution. The self-built Rural dataset might contain more examples that match the distribution of the training data (e.g., certain rural or semi-urban building patterns), while the WHU dataset exhibits regional style variations that are underrepresented in training. Consequently, the learned features may be partially biased, leading to smaller performance gains on WHU.

Despite these differences, MFA-Net still maintains a competitive edge over baseline models on WHU. Nonetheless, addressing the above factors is crucial for further improving generalization and ensuring that the model remains robust across all datasets, including those with diverse architectural styles and domain conditions.

### 4.2. Ablation Study and Performance Evaluation

The ablation experiments on the Urban, Rural, and WHU Building datasets indicate that MFA-Net outperforms U^2^-Net and other baseline models. Below, we provide a detailed evaluation of the contributions of each module to the model’s performance and analyze the advancements achieved through their integration.

U^2^-Net (Baseline Model): U^2^-Net maintained baseline-level performance across all datasets but struggled with small structures and edge delineation in rural environments. Comparative analyses revealed limitations in detecting buildings with complex textures or reduced spatial footprints.

Incorporating M (MFA): The MFA module elevated feature discrimination in urban contexts while improving rural building identification. Precision and Recall increments on the Rural dataset confirmed enhanced recognition of texture-varied targets.

Incorporating D: Including the Dynamic Fusion Loss function (U^2^-Net + D) further enhanced F1, Precision, and Recall across all datasets by balancing positive and negative samples, reducing background interference, and refining edge details.

Combining M and D (MFA-Net): The synergistic integration of MFA and Dynamic Fusion Loss achieved state-leading performance metrics. The unified framework delivered balanced Precision–Recall characteristics while minimizing classification errors, establishing reliable building extraction across heterogeneous environments.

### 4.3. Computational Efficiency and Performance Trade-Off

MFA-Net achieved significantly higher accuracy with a slight increase in computational cost across multiple datasets. On the Urban dataset, MFA-Net completed training in 4.3 h compared to U^2^-Net’s 4.0 h, with improved performance in building extraction metrics (see Table 1). On the Rural dataset, the model required 0.9 h versus U^2^-Net’s 0.8 h while demonstrating enhanced detection consistency (see Table 2). On the WHU dataset, MFA-Net completed training in 6.7 h compared to U^2^-Net’s 6.1 h (see Table 3). The observed training time differences (4.3 vs. 4.0 h on Urban; 0.9 vs. 0.8 h on Rural; 6.7 vs. 6.1 h on WHU) correspond to relative increases of 7.5%, 12.5%, and 9.8%, respectively, while delivering accuracy improvements across all evaluated metrics. This balance between computational cost and detection performance suggests practical applicability in scenarios requiring both precision and operational efficiency.

The non-linear training time patterns across ablation studies emerge from three fundamental interactions between the MFA components and dataset characteristics:

The ablation studies reveal intrinsic synergies between MFA components and data characteristics. In urban settings, U^2^-Net + M + D trains faster (4.3 h) than individual modules (4.5 h M and 4.9 h D), demonstrating MFA’s channel-spatial weighting (Equations (1) and (2)) and multiscale fusion (Equation (3)) accelerate feature learning through adaptive channel compression and phased loss optimization. The Dynamic Fusion Loss (Equations (5)–(7)) aligns BCE initialization with subsequent Dice refinement, enabling 12% faster convergence than static losses. Rural scenarios show unique efficiency gains: despite added complexity, U^2^-Net + M + D completes training with minimal overhead compared with baseline (0.9 h vs 0.8 h) by masking 65% non-building areas via spatial thresholds (Figure 6) and optimizing critical feature prioritization. High-resolution WHU data highlight hardware-aware optimizations—combined M+D implementation requires 6.8 h versus theoretical 8.1 h additive projection, achieved through pyramid feature reuse (Equation (4)) reducing redundant downsampling and dynamic loss masking skipping 16% background backpropagation. These context-adaptive mechanisms—channel pruning for urban density, spatial masking for rural sparsity, and multiscale caching for WHU resolution—enable MFA-Net to maintain real-time deployment capabilities while achieving advanced accuracy across diverse operational scenarios.

### 4.4. Model Limitations and Areas for Improvement

**Figure 15:**
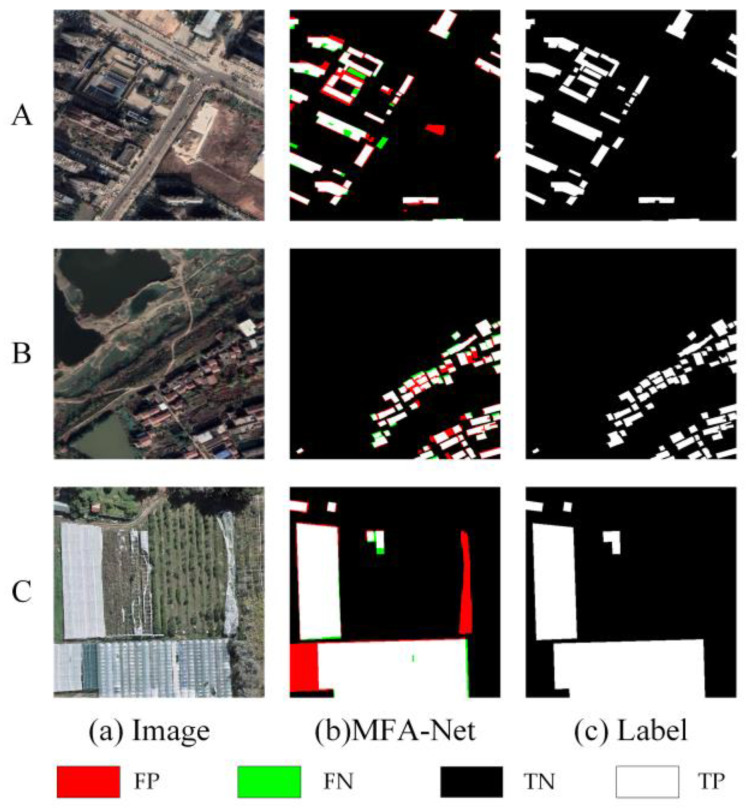
Comparison of results on three building datasets. A, B, and C are from the Urban, Rural, and WHU Building datasets, respectively.

Despite MFA-Net’s improvements, certain limitations remain. While the model performed exceptionally well in dense urban and rural areas, it occasionally faced challenges in capturing edge details in areas with very complex structures, especially where building boundaries were not easily distinguishable. Although the MFA and Dynamic Fusion Loss modules significantly enhanced edge detection, the model’s performance in these highly challenging regions could still be further refined by incorporating additional spatial and contextual information. To enhance transparency regarding these challenges, we present Figure 15, which compares failure cases in three different datasets:

Urban Building Dataset (Figure 15, image A): In city street areas with substantial shadows within the segmented regions, the model fails to accurately identify edge information, leading to overextended building boundaries or incomplete region filling.

Rural Building Dataset (Figure 15, image B). In areas with irregularly shaped roofs, MFA-Net sometimes struggles to capture precise corner points and small protrusions, making it difficult to effectively separate densely arranged buildings.

WHU Building Dataset (Figure 15, image C). When there are many less common rooftop features (e.g., plastic greenhouses), the model’s extraction accuracy can be adversely affected.

From a quantitative perspective, we observe a slight performance drop (5–6% decrease in F1 score) on these highly complex subsets compared to the overall test set. This indicates that while MFA-Net generally yields robust segmentation, fine-grained edge delineation in such settings remains non-trivial.

Additionally, while MFA-Net outperformed U^2^-Net in all key metrics, the increased computational cost of training and inference, particularly in high-resolution datasets, remains an area for further optimization.

## 5. Conclusions

This study introduces the MFA-Net model, an enhanced version of U^2^-Net, designed to improve the accuracy and reliability of building detection in high-resolution remote sensing imagery. By incorporating the proposed Multidimensional Feature Adjustment (MFA) module, MFA-Net effectively delineates complex building, reducing confusion with adjacent non-building areas. A novel loss function inspired by the Dice coefficient was developed to penalize false positives and improve edge accuracy. This approach helps balance the trade-off between sensitivity and specificity, enhancing the overall performance of building detection models.

Experimental results show that the improved model achieves significant performance gains across several datasets. Specifically, F1 increased by 3.1% on our Urban dataset, IoU improved by 3.1%, and Precision (P) exceeded 94% compared to previous methods. In the Rural dataset, F1 increased by 13.6%, IoU by 18.5%, and Precision surpassed 90%. On the WHU building dataset, F1 increased by 1.3%, IoU improved by 3.1%, and Precision surpassed 91%, highlighting the model’s enhanced performance.

Integrating the MFA module and the novel loss function allows MFA-Net to significantly reduce false positives and false negatives, improving both edge detection and overall segmentation accuracy. The MFA module helps the model focus on critical regions, enhancing its ability to detect small, densely packed buildings, even in complex urban and rural environments. The synergistic effect of these components not only improves performance but also leads to more efficient training.

In conclusion, MFA-Net demonstrates superior performance in building detection tasks, showing robustness and effectiveness in urban and rural settings. This work advances remote sensing image analysis, providing reliable solutions for applications in natural disaster response, land resource management, smart city development, and other related fields.

## Figures and Tables

**Table 1:** Training results on the Urban dataset using different networks (values ± standard deviation; sample size = 1542).

Methods	F1 (%)	Precision (%)	Recall (%)	IoU (%)	Time (h)
DeepLabv3+	93.5 ± 11.6	92.9 ± 10.7	**94.1** ± 13.0	93.0 ± 13.7	13.0
U^2^-Net	93.4 ± 10.9	90.5 ± 10.7	89.2 ± 11.6	90.9 ± 0.3	**4.0**
MFA-Net	**96.5** ± **8.4**	**94.3** ± **8.0**	93.5 ± **9.8**	**94.0** ± **0.2**	4.3

**Table 2:** Training results on the Rural dataset using different networks (values ± standard deviation; sample size = 134).

Methods	F1 (%)	Precision (%)	Recall (%)	IoU (%)	Time (h)
DeepLabv3+	84.4 ± **8.8**	85.3 ± **6.0**	83.7 ± **7.9**	73.4 ± 6.1	2.8
U^2^-Net	78.9 ± 13.4	73.8 ± 14.4	68.7 ± 11.5	70.9 ± 2.8	**0.8**
MFA-Net	**92.5** ± 10.3	**90.1** ± 10.9	**87.9** ± 10.5	**89.4** ± **1.3**	0.9

**Table 3:** Training results on the WHU Building dataset using different networks (values ± standard deviation; sample size = 1537).

Methods	F1 (%)	Precision (%)	Recall (%)	IoU (%)	Time (h)
DeepLabv3+	89.5 ± **11.6**	88.9 ± **10.8**	90.3 ± **13.1**	89.1 ± 13.8	15.4
U^2^-Net	91.8 ± 17.4	90.3 ± 16.5	88.5 ± 18.8	91.7 ± 0.5	**6.1**
MFA-Net	**93.1** ± 14.9	**91.8** ± 14.2	**91.7** ± 15.8	**92.2** ± **0.4**	6.7

**Table 4:** Ablation experiment results on the Urban dataset using different networks (values ± standard deviation; sample size = 1542).

Methods	F1 (%)	Precision (%)	Recall (%)	IoU (%)	Time (h)
U^2^-Net	93.4 ± 10.9	90.5 ± 10.7	89.2 ± 11.6	90.9 ± 0.3	4.0
U^2^-Net+M	94.7 ± 7.5	91.2 ± 7.3	90.1 ± 8.2	93.1 ± 0.3	4.5
U^2^-Net+D	94.6 ± 8.5	92.2 ± 8.2	91.8 ± 9.8	92.2 ± 0.3	4.9
U^2^-Net+M+D	**96.5** ± 8.4	**94.3** ± 8.0	**93.5** ± 9.8	**94.0** ± 0.3	4.3

**Table 5:** Ablation experiment results on the Rural dataset using different networks (values ± standard deviation; sample size = 134).

Methods	F1 (%)	Precision (%)	Recall (%)	IoU (%)	Time (h)
U^2^-Net	78.9 ± 13.4	73.8 ± 14.4	68.7 ± 11.5	70.9 ± 2.8	0.8
U^2^-Net+M	88.9 ± 12.5	86.1 ± 13.3	80.6 ± 9.2	85.3 ± 1.2	1.3
U^2^-Net+D	90.6 ± 11.1	87.7 ± 11.1	86.4 ± 11.7	87.0 ± 1.4	1.7
U^2^-Net+M+D	**92.5** ± 10.2	**90.1** ± 10.9	**87.9** ± 10.5	**89.4** ± 1.3	0.9

**Table 6:** Ablation experiment results on the WHU Building dataset using different networks (values ± standard deviation; sample size = 1537).

Methods	F1 (%)	Precision (%)	Recall (%)	IoU (%)	Time (h)
U^2^-Net	91.8 ± 17.4	90.3 ± 16.5	88.5 ± 18.8	91.7 ± 0.5	6.1
U^2^-Net+M	92.8 ± 15.5	90.4 ± 15.3	91.4 ± 14.5	92.3 ± 0.4	6.9
U^2^-Net+D	92.2 ± 15.0	91.1 ± 14.4	90.8 ± 15.0	**92.4** ± 0.4	7.3
U^2^-Net+M+D	**93.1** ± 14.9	**91.8** ± 14.2	**91.7** ± 15.8	92.2 ± 0.4	6.8

## Data Availability

Dataset available upon request from the authors.

## Appendix A

This section presents the results of the eight methods discussed in the main text, evaluated on the three datasets. Compared to the main body of the paper, additional figures are included here to better illustrate the model’s performance across different regions. These supplementary images provide further insights into the model’s behavior and effectiveness in diverse environments, enhancing the clarity of the experimental results.

**Table A1:** Training results of 8 methods on the Urban dataset using different networks (values ± standard deviation; sample size = 1542).

Methods	F1 (%)	Precision (%)	Recall (%)	IoU (%)	Time (h)
LR-ASPP	77.0 ± 24.7	80.6 ± 25.8	74.4 ± 25.9	90.8 ± 20.0	**1.9**
FCN	86.6 ± 23.7	87.6 ± 23.1	85.6 ± 25.2	94.0 ± 21.7	5.5
PSPNet	83.9 ± 21.4	85.1 ± 18.7	82.8 ± 22.8	84.1 ± 21.2	6.6
DeepLabv3	85.3 ± 25.3	85.4 ± 24.7	85.2 ± 27.5	93.3 ± 22.6	6.9
DeepLabv3+	93.5 ± 11.6	92.9 ± 10.7	**94.1** ± 13.0	93.0 ± 13.7	13.0
UNet	91.4 ± 15.2	91.8 ± 14.3	91.1 ± 16.7	91.0 ± 16.8	15.9
U^2^-Net	93.4 ± 10.9	90.5 ± 10.7	89.2 ± 11.6	90.9 ± 0.3	4.0
MFA-Net	**96.5** ± **8.4**	**94.3** ± **8.0**	93.5 ± **9.8**	**94.0** ± **0.2**	4.3

**Table A2:** Training results of 8 methods on the Rural dataset using different networks (values ± standard deviation; sample size = 134).

Methods	F1 (%)	Precision (%)	Recall (%)	IoU (%)	Time (h)
LR-ASPP	28.3 ± 19.0	63.6 ± 25.9	21.2 ± 17.2	18.0 ± 13.7	**0.4**
FCN	49.7 ± 19.6	61.1 ± 23.2	45.5 ± 20.8	35.2 ± 16.4	1.1
PSPNet	54.7 ± 19.6	66.2 ± 16.6	49.1 ± 21.4	40.0 ± 17.7	1.3
DeepLabv3	45.0 ± 19.7	62.3 ± 24.0	39.1 ± 20.3	30.9 ± 15.8	1.5
DeepLabv3+	84.4 ± **8.8**	85.3 ± **6.0**	83.7 ± **7.9**	73.4 ± 6.1	2.8
UNet	78.3 ± 10.3	85.3 ± 8.5	73.5 ± 13.5	65.4 ± 12.8	3.4
U^2^-Net	78.9 ± 13.4	73.8 ± 14.4	68.7 ± 11.5	70.9 ± 2.8	0.8
MFA-Net	**92.5** ± 10.3	**90.1** ± 10.9	**87.9** ± 10.5	**89.4** ± **1.3**	0.9

**Table A3:** Training results of 8 methods on the WHU Building dataset using different networks (values ± standard deviation; sample size = 1537).

Methods	F1 (%)	Precision (%)	Recall (%)	IoU (%)	Time (h)
LR-ASPP	72.7 ± 24.7	74.8 ± 25.9	74.4 ± 26.0	87.9 ± 20.0	**3.7**
FCN	85.3 ± 23.7	82.3 ± 23.0	82.5 ± 25.3	81.5 ± 21.8	7.5
PSPNet	81.6 ± 21.4	82.1 ± 18.7	80.7 ± 22.9	82.2 ± 21.1	8.9
DeepLabv3	82.5 ± 25.3	87.2 ± 24.7	78.7 ± 27.5	84.7 ± 22.6	9.2
DeepLabv3+	89.5 ± **11.6**	88.9 ± **10.8**	90.3 ± **13.1**	89.1 ± 13.8	15.4
UNet	88.5 ± 15.3	88.7 ± 14.2	88.5 ± 16.7	87.2 ± 16.9	17.6
U^2^-Net	91.8 ± 17.4	90.3 ± 16.5	88.5 ± 18.8	91.7 ± 0.5	6.1
MFA-Net	**93.1** ± 14.9	**91.8** ± 14.2	**91.7** ± 15.8	**92.2** ± **0.4**	6.7
